# Advanced Fabrication Method and Mechanical Properties of Silicon Nitride/Boron Nitride Fibrous Monolithic Ceramics

**DOI:** 10.3390/ma16186130

**Published:** 2023-09-08

**Authors:** Qingqing Chen, Yuan Zhang, Liuxin Chao, Ningning Dong, Yu Zhou, Guobing Ying

**Affiliations:** 1 Department of Materials Science and Engineering, College of Mechanics and Materials, Hohai University, Nanjing 211100, China; 2 Institute for Advanced Ceramics, School of Materials Science and Engineering, Harbin Institute of Technology, Harbin 150001, China; 3 Key Laboratory of Advanced Structural-Functional Integration Materials & Green Manufacturing Technology, Harbin Institute of Technology, Harbin 150001, China; 4 School of Materials Science and Engineering, Harbin Institute of Technology (Shenzhen), Shenzhen 518055, China

**Keywords:** Si_3_N_4_/BN, fibrous monolithic ceramic, microstructure, fracture toughness

## Abstract

Silicon nitride ceramics are regarded as a promising material for high-temperature structural applications due to their remarkable characteristics, including high strength, hardness, thermal conductivity, low dielectric properties, and resistance to creep at elevated temperatures. However, their susceptibility to catastrophic fracture at high temperatures remains a concern. Herein, Si_3_N_4_/BN fibrous monolithic ceramics have been successfully prepared by employing wet-spinning and hot-pressing techniques. We delved into the design and optimization of the spinning slurry and examined how the Si_3_N_4_/BN fiber diameter affects the ceramics’ microstructure and mechanical properties. The spinning slurry exhibited exceptional stability and spinnability. Decreasing the fiber diameter contributed to material densification and improved mechanical properties. Notably, when the fiber diameter is 0.9 mm, the fabricated Si_3_N_4_/BN fibrous monolithic ceramics demonstrate a carbon content of 0.82%, a three-point bending strength of 357 ± 24 MPa, and a fracture toughness of 8.8 ± 0.36 MPa·m^1/2^. This investigation offers valuable insights into producing high-performance Si_3_N_4_/BN composite ceramics utilizing hot-pressing technology.

## 1. Introduction

The deterioration of the service environment necessitates more stringent requirements for radomes on high Mach number aircraft, which are essential for maintaining structural integrity and enabling precise communication guidance. Silicon nitride (Si_3_N_4_) demonstrates excellent transparency to electromagnetic waves, along with notable properties such as high strength, high temperature stability, and exceptional resistance to oxidation [1,2,3,4]. Wave transparency primarily relies on the inherent composition and structure of the material system [5,6,7,8], which can be achieved through meticulous material preparation processes. However, enhancing the mechanical properties of wave-transparent materials poses challenges, often requiring optimization of interface structures and the achievement of congruence between the structure and properties within the composite constituents.

Si_3_N_4_/BN fibrous monolithic ceramics comprise a principal phase (Si_3_N_4_ units) and an interfacial phase (BN unit interfaces), and are known for their strength, wave transparency, chemical stability, and temperature resilience [9,10,11,12,13,14,15,16,17]. Notably, cracks in these ceramics tend to propagate along the unit interfaces, requiring higher fracture energy, such as through crack deflection or branching. Maximizing these mechanical properties is crucial for expanding the applications of Si_3_N_4_/BN fibrous monolithic ceramics.

Kiyoshi [18] proposed an innovative method for precise control of the microstructure of self-reinforced silicon nitride. This novel approach involves using a ribbon casting film to arrange rod-like single crystal seeds of *β*-Si_3_N_4_, effectively controlling the size and distribution of fibrous large grains. Consequently, the fracture toughness of silicon nitride ceramics prepared using this method is significantly improved, reaching 11.1 MPa·m^1/2^. Previous studies primarily focused on incorporating *β*-Si_3_N_4_ rod-like seeds [9,10] or SiC whiskers [14,15,16] into Si_3_N_4_ fibers. However, the debinding process in these studies was conducted in an inert atmosphere, which, while enhancing the mechanical properties of Si_3_N_4_/BN fibrous monolithic ceramics, adversely affects their transparency to waves.

This research aims to delve into the relationship between the wet-spinning–hot-pressing sintering process and the microstructure and mechanical properties of Si_3_N_4_/BN fibrous monolithic ceramics. We produced Si_3_N_4_/BN fibrous monolithic ceramics utilizing the wet-spinning–hot-pressing sintering technique, investigating their microstructure and mechanical properties. Our focus was on stabilizing the wet-spinning slurry by adjusting the solid content and sodium alginate content, without adding *β*-Si_3_N_4_ rod-shaped seeds or SiC whiskers. The debinding process was conducted in an ambient air environment to minimize residual carbon. We also assessed how fiber diameter (ranging from 0.42 to 1.05 mm) influences the ceramics’ microstructure and mechanical properties. These findings offer a valuable strategy for designing and optimizing the structure of fibrous monolithic ceramics to enhance their overall performance.

## 2. Materials and Methods

### 2.1. Materials and Formulation of Spinning Solutions

To promote fiber formation and avert crack formation during drying, sodium alginate (CP grade, Shanghai National Pharmaceutical Reagent Group, Shanghai, China) was incorporated as a binder in the spinning solution. The sodium alginate binder was dissolved in deionized water, and the initial powder was combined with the sodium alginate aqueous solution through 12 h of planetary ball milling, ensuring adequate particle penetration and obtaining the spinning solution. The proportions of the initial powders are presented in Table 1, consistent with our previous work [19]. By homogenously mixing sodium alginate aqueous solutions with different mass fractions (2.5 wt.%, 3.0 wt.%, and 3.5 wt.%) with 50 wt.% initial powder, the corresponding spinning solutions (P4, P2, and P5) were generated. Furthermore, based on P2, spinning solutions P1 and P3 were prepared by substituting the 50 wt.% initial powder with 40 wt.% and 60 wt.% initial powder, respectively. The compositions of the spinning solutions are presented in Table 2.

### 2.2. Rheological Properties of Spinning Solution and TG Analysis

The rheological behavior of the spinning precursor solution at ambient temperature was characterized using an MCR301 rotational rheometer (Graz, Austria), with a plate–plate geometry of 8 mm diameter and a gap of 2 mm. A constant frequency of 1 Hz was applied during stress scans, and oscillatory shear experiments were performed in the range of 1 to 10 with a 5 Pa amplitude. The apparent viscosity of the spinning precursor solution was recorded during strain scans from 0.1 to 10 L/s. The thermal stability of the spinning precursor solution was investigated in air, from room temperature to 1000 °C, using thermogravimetric analysis (TGA, TGA2050, Houston, TX, USA) with a heating rate of 15 °C/min.

### 2.3. Sample Spinning, Drying, and Sintering

**Figure 1:**
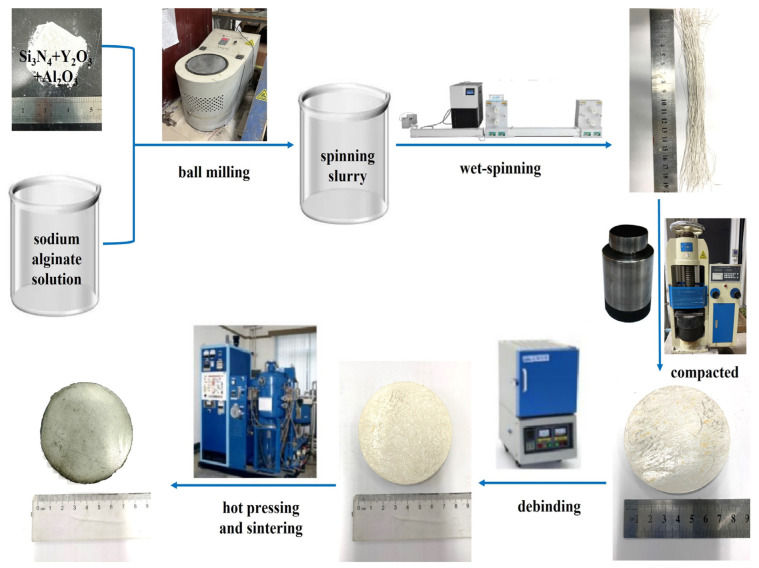
Schematic diagram of Si_3_N_4_/BN fibrous monolithic ceramic preparation.

The process flowchart of Si_3_N_4_/BN fibrous monolithic ceramics is depicted in Figure 1. The process begins by weighing BN powder and dispersing it in deionized water to prepare a 5 wt.% BN slurry. The spinning solution is then introduced into a syringe and extruded through a tube connected to the needle into an anhydrous CaCl_2_ hydrogel bath, where the pressure of the injection pump induces rapid solidification of the Si_3_N_4_ slurry into ceramic fibers. The solidified fibers are then coated with the BN interfacial slurry by being drawn into the BN slurry pool through roller rotation. After drying at room temperature, the fibers are cut into short segments of a specific size and arranged parallel in a chromium steel mold. The assembly is pre-pressed at 20 MPa for 5 min and then heated at a rate of 1 °C/min to 600 °C in a muffle furnace to remove sodium alginate. Pure Si_3_N_4_/BN fibrous monolithic ceramics are obtained through hot pressing. This is performed in a graphite mold containing a powder bed of 50 wt.% BN and 50 wt.% Si_3_N_4_. The samples are heated to 1800 °C at a rate of 15 °C/min and held at 28 MPa for 2 h.

### 2.4. Testing Methods

The microstructure of Si_3_N_4_/BN fibrous monolithic ceramics was scrutinized using a HITACHI S4800 scanning electron microscope (SEM). To supplement this, an energy dispersive spectrometer (EDS) was utilized for an in-depth analysis. The linear contraction rate was ascertained by measuring the dimensional changes between the gel and the bulk post-casting. For a comprehensive chemical composition analysis, the Si_3_N_4_/BN fibrous monolithic ceramic samples were ground and assessed for carbon elements using the Leco CS-200 carbon-sulfur analyzer.

Mechanical properties, specifically the three-point flexural strength and fracture toughness of Si_3_N_4_/BN fibrous monolithic ceramics, were evaluated at room temperature using the INSTRON-3367 universal testing machine (Instron Group, Norwood, MA, USA). To prepare for this, ceramic blocks underwent a series of processes: they were cut using an internal circular cutting machine, ground, polished to achieve a mirror finish, chamfered, and cleaned. This resulted in test samples of dimensions 3 × 4 × 36 mm and 2 × 4 × 22 mm.

For the flexural strength assessment, 3 × 4 × 36 mm specimens were selected. The tests were conducted with a 30 mm span and a loading speed of 0.5 mm/min. Results from 4–6 samples were averaged. The flexural strength was calculated using Formula (1):

(1) $$\sigma =\frac{3PL}{2BW^{2}}$$

where *σ* represents bending strength (MPa); *P* is the maximum applied load (N); *L* is the span during testing (30 mm); *B* is the width (approximately 4 mm); and *W* is the height (approximately 3 mm).

Fracture toughness was determined following the GB/T 23806-2009 standards [15], using 2 × 4 × 20 mm specimens and the single-edge notched beam method. The notch depth was kept under 2 mm. During testing, the maximum fracture load was recorded, the notch depth was measured, and the press head’s movement rate was set at 0.05 mm/min with a 16 mm span. Results from 4–6 samples were averaged. The fracture toughness was calculated using Formula (2):

(2) $$K_{IC}=\frac{3PL\sqrt{a\times 10^{-3}}}{2BW^{2}}\left[1.93-3.07\left(\frac{a}{W}\right)+14.53{\left(\frac{a}{W}\right)}^{2}-25.11{\left(\frac{a}{W}\right)}^{3}+25.8{\left(\frac{a}{W}\right)}^{4}\right]$$

where *K_IC_* is the fracture toughness (MPa·m^1/2^); *P* is the maximum applied load (N); *L* is the span (16 mm); a is the notch depth (mm); *B* is the width (approximately 2 mm); and *W* is the height (approximately 4 mm).

## 3. Results

### 3.1. Rheological Properties

The rheological properties of the spinning slurry are vital for determining the quality of wet spinning. These properties are typically assessed using three parameters: modulus, yield stress, and apparent viscosity. For optimal wet spinning, the slurry should exhibit shear-thinning behavior, with an ideal storage modulus (~10^5^ Pa) and yield stress (10^2^–10^3^ Pa) under high shear conditions [11]. The storage modulus is indicative of the extrudability of the slurry, which is its ability to be smoothly extruded from the nozzle under appropriate shear stress. On the other hand, the yield stress reflects the slurry’s spinnability, its capacity to retain structural stability and support the structures formed post-extrusion. The rheological properties are significantly influenced by solid mass fractions, offering insights for the preparation and optimization of spinning slurry. To obtain the desirable extrudability and spinnability, we evaluated slurries with distinct solid mass fractions (40 wt.% (P1), 50 wt.% (P2), and 60 wt.% (P3)) to evaluate their rheological properties.

**Figure 2:**
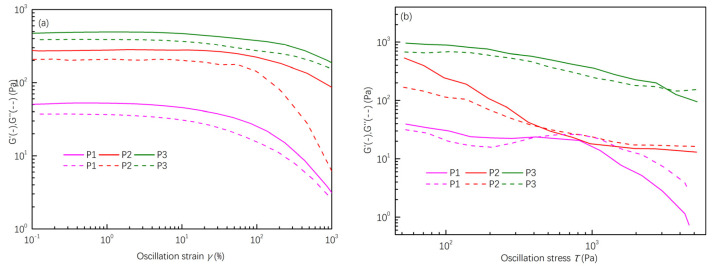
Variation of storage modulus (*G*′) and loss modulus (*G*″) over strains (**a**) and stress (**b**) of spinning slurries with different solid mass fractions.

**Figure 3:**
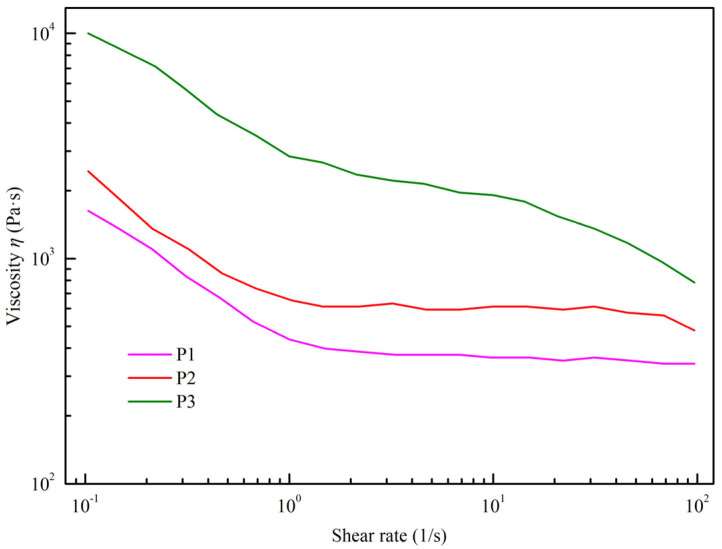
The steady shear viscosity of spinning slurries with different solid mass fraction.

**Figure 4:**
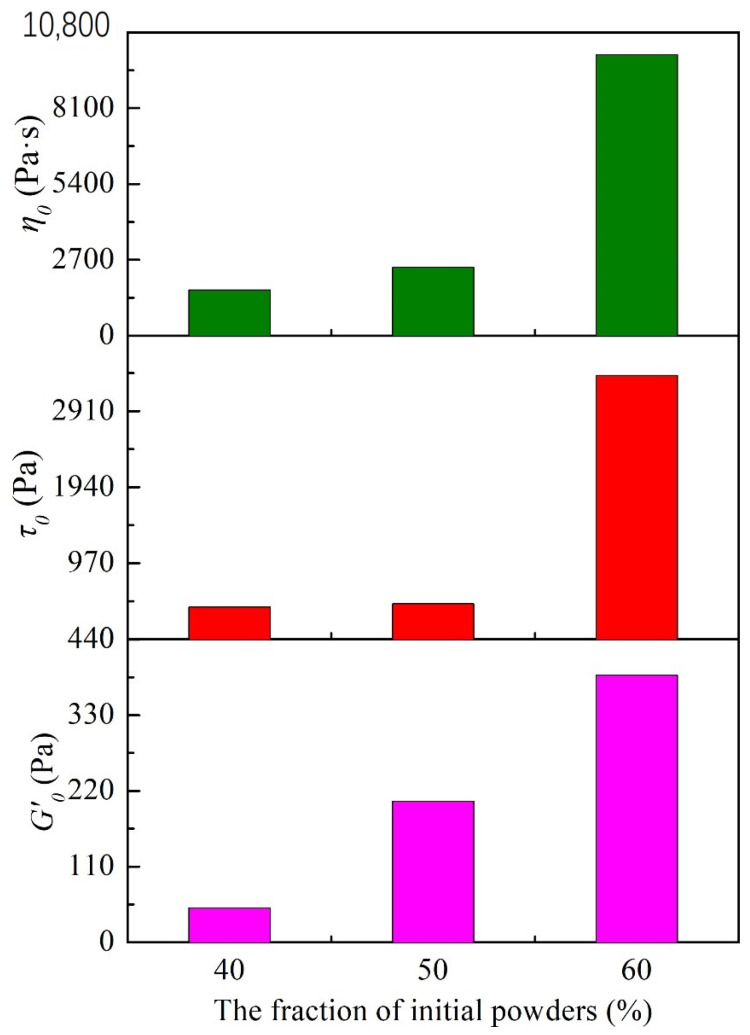
The initial storage modulus (*G*′_0_), yield stresses (*τ*_0_) and initial apparent viscosity (*η*_0_) of spinning slurries with different solid mass fraction.

The impact of different solid mass fractions on the rheological properties is demonstrated in Figure 2, Figure 3 and Figure 4. The *G*′/*G*″-*γ* curve (Figure 2a) shows that the storage modulus (*G*′) surpasses the loss modulus (*G*′) under the same shear strain, indicating viscoelastic behavior. However, with increasing shear stress, the two curves intersect, leading the loss modulus (*G*″) to exceed the storage modulus (*G*′). This indicates that the slurry samples can be extruded into shape with the right extrusion pressure. Nevertheless, an increase in the solid mass fraction significantly boosts both the storage (*G*′) and loss modulus (*G*″) of the slurry. For instance, at a solid mass fraction of 60 wt.% (P3), the storage modulus (*G*′) reaches approximately 388 Pa. This higher modulus necessitates greater extrusion pressure, increasing the risk of nozzle clogging and printing interruption. On the other hand, slurries with lower solid mass fractions (P1) have a reduced modulus, increasing the chances of splatter during spinning and affecting their self-supporting ability.

The steady-state shear test results for slurries with different solid mass fractions are illustrated in Figure 3. All samples demonstrate shear-thinning behavior, with their apparent viscosities decreasing at elevated shear rates, underscoring the non-Newtonian characteristics of the slurries. Notably, the initial apparent viscosity rises significantly with an increase in solid mass fractions, jumping from 1.6 × 10^3^ Pa·s in P1 to 1.0 × 10^4^ Pa·s in P3. According to Woodcock’s Formula (3), when the particle diameter (*d*) remains constant, the distance (*h*) between particles is inversely related to the solid mass fraction [12]. This implies that as the solid content increases, the likelihood of particle contact also rises. This amplifies the interparticle van der Waals forces and enhances particle flocculation within the slurry. Consequently, during shearing, there is an uptick in frictional forces between particles, leading to elevated moduli (*G*) and apparent viscosities (*η*).

(3) $$\frac{h}{d}={\left(\frac{1}{3\pi \emptyset }+\frac{5}{6}\right)}^{1/2}-1$$

The statistical results for the initial storage modulus (*G*′_0_) and yield stress (*τ*_0_) are presented in Figure 4. Compared to the other samples, the initial storage modulus (*G*′_0_) of the P1 (40 wt.%) is significantly lower, along with a relatively small yield stress (*τ*_0_). The P2 (50 wt.%) has a storage modulus of approximately 205 Pa and a corresponding yield stress (*τ*_0_) of 455 Pa, both of which are within a suitable range for extrusion and indicate excellent self-supporting capability. However, the P3 slurry (60 wt.%) demonstrates a storage modulus close to 388 Pa, while the yield stress reaches 3366 Pa, making it unsuitable for wet-spinning applications. Based on the analysis mentioned above, neither the P1 slurry nor the P3 slurry is suitable for wet spinning.

**Figure 5:**
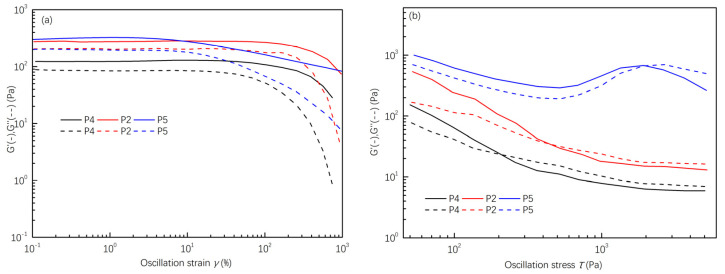
Variation of storage modulus (*G*′) and loss modulus (*G*″) over strains (**a**) and stress (**b**) of spinning slurries with different proportions of sodium alginates.

**Figure 6:**
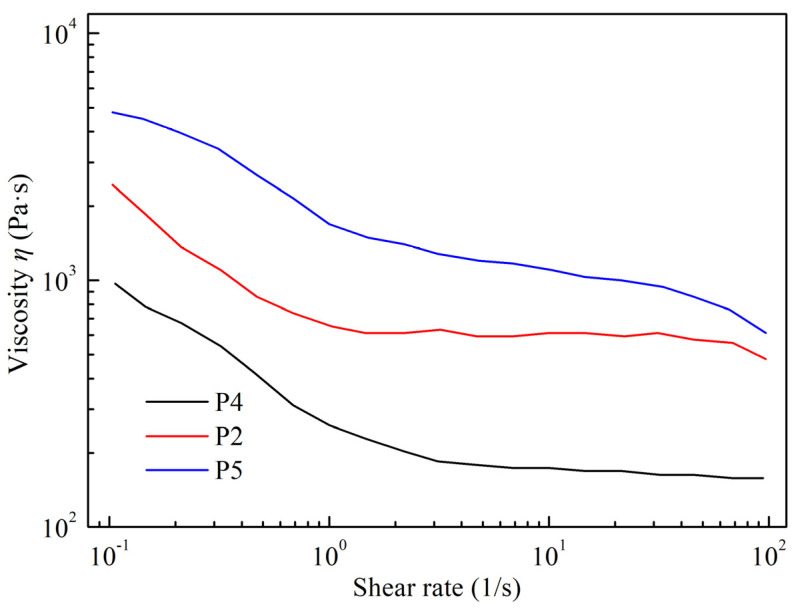
The steady shear viscosity of spinning slurries with proportions of sodium alginates.

**Figure 7:**
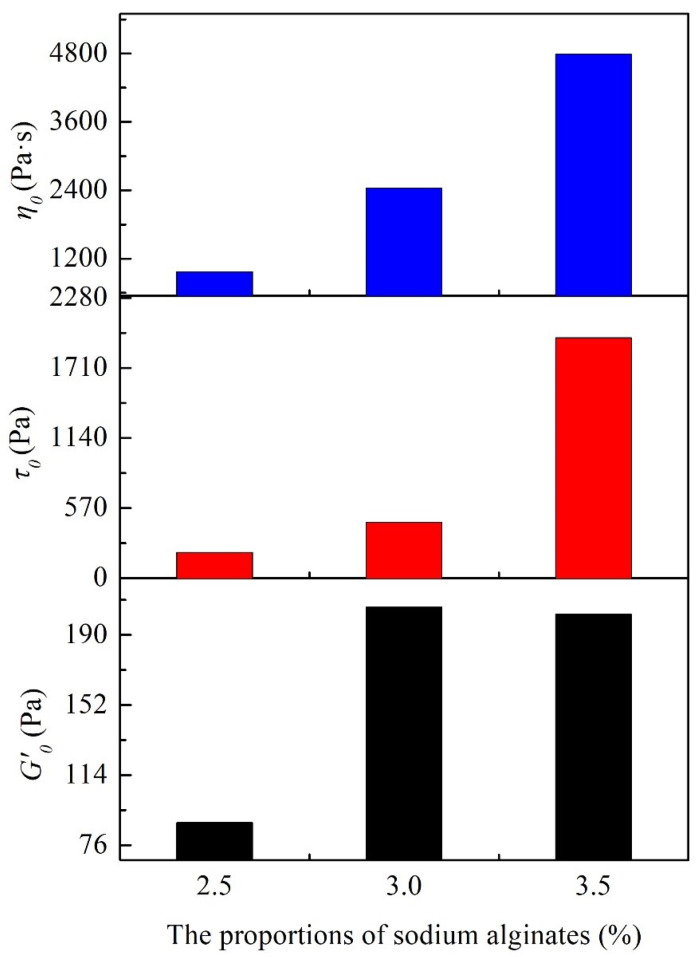
The initial storage modulus (*G*′_0_), yield stress (*τ*_0_), and initial apparent viscosity (*η*_0_) of spinning slurries with proportions of sodium alginates.

Fiber-reinforced cordierite ceramics are susceptible to cracks due to shrinkage stress during drying. To counteract this, hydrophilic polymers with abundant hydroxyl groups (–OH) can crosslink and reinforce the green body via hydrogen bonding. Consequently, they are employed in gel casting to prevent cracking and skinning of the green body [11]. Sodium alginate, a non-toxic and cost-effective water-soluble polymer, demonstrates strong binding capabilities. Even in small quantities, it can significantly enhance the properties of the green body. Additionally, sodium alginate decomposes oxidatively at lower temperatures, ensuring no residual carbon remains in the matrix. For optimal ceramic green bodies, a solution with a solid mass fraction of 50 wt.% was prepared by introducing sodium alginate in varying proportions (2.5 wt.% (P4), 3.0 wt.% (P2), and 3.5 wt.% (P5)). The rheological properties of samples with different sodium alginate contents are depicted in Figure 5, Figure 6 and Figure 7. Specifically, Figure 5a shows that the samples containing sodium alginate exhibit higher storage modulus (*G*′) values than the loss modulus (*G*″) at minimal shear strains, indicating viscoelastic behavior. Figure 5b reveals the point of yield stress (*τ*_0_) where the storage modulus (*G*′) intersects with the loss modulus (*G*″) under increasing shear stress, implying that the slurry containing sodium alginate can transition into a liquid sol under appropriate pressure.

The steady-state shear test results of spinning slurries with different sodium alginate proportions are shown in Figure 6. The initial apparent viscosity of the slurry increases with increasing sodium alginate content. Sodium alginate introduces a significant number of hydroxyl (–OH) groups. These groups enable sodium alginate molecules to crosslink with silane groups (≡Si–NH–Si≡, ≡Si–NH_2_), silanol groups (≡Si–OH), and other sodium alginate molecules on the surfaces of ceramic particles through hydrogen bonding [12]. As a result, ceramic particles within the slurry are arranged in a three-dimensional colloidal network structure, facilitated by sodium alginate molecules. Adding sodium alginate enhances the slurry’s binding force. With more sodium alginate, the molecular bridging effect becomes pronounced, restricting particle movement. This makes the transition from a solid gel to a liquid sol during shearing more resistant. Nevertheless, in practical wet-spinning processes, the pressure exerted by the wet-spinning equipment is limited, which can hinder the even extrusion of a high-viscosity slurry, affecting material deposition. Therefore, the amount of sodium alginate introduced should be controlled. The comparison of the initial storage modulus (*G*′_0_), yield stress (*τ*_0_), and initial apparent viscosity (*η*_0_) among samples P4, P2, and P5 (Figure 7) indicates an upward trend in the initial storage modulus and yield stress as the sodium alginate content increases. The yield stresses are 208 Pa (P4), 455 Pa (P2), and 1955 Pa (P5), respectively. Given this, sample P5, with a yield stress exceeding 103 Pa, is not suitable for wet-spinning slurry extrusion.

In summary, P2 is suitable for slurry extrusion in wet spinning, demonstrating an excellent self-supporting capability with a yield stress value of 455 Pa. Consequently, the P2 with moderate rheological properties was selected for further investigation.

### 3.2. Drying and Defoaming Processes for Green Bodies

#### 3.2.1. Drying Process for Green Bodies

**Figure 8:**
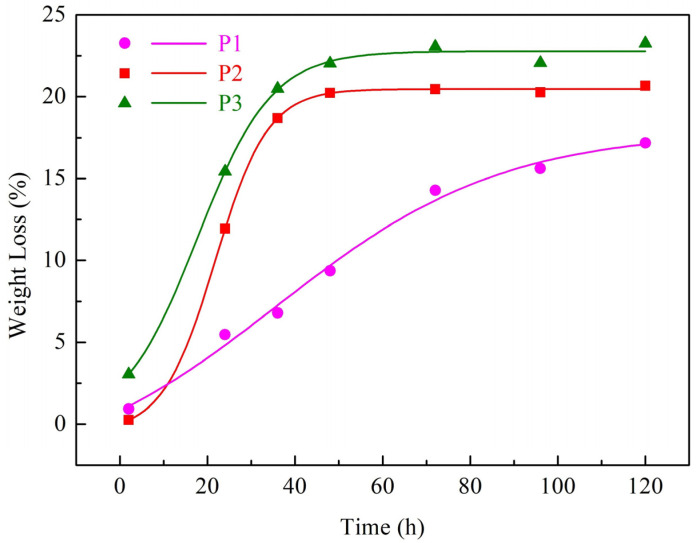
The relative weight loss curves of ceramic green bodies based on Si_3_N_4_/BN fibrous monolithic ceramics with different mass fractions.

The drying treatment is pivotal for the formation of Si_3_N_4_/BN fiber-reinforced monolithic ceramics. This is due to the profound influence of moisture loss on the internal microstructure of green bodies. If not dried adequately, the green body can peel or crack during sintering, compromising the final product’s quality. Figure 8 illustrates the relative weight loss curves of silicon nitride-based ceramic green bodies with different solid mass fractions. The initial drying stage demonstrates the highest rate of sample weight loss, which then significantly decreases until reaching a stable final weight after 48 h. The overall relative weight loss rate of the sample exhibits a trend of slowing down with time. The overall relative weight loss rate of the sample exhibits a diminishing trend over time. Furthermore, Figure 8 demonstrates that a higher solid mass fraction leads to a decreased relative weight loss per unit time for the sample. This can be attributed to the decrease in water content as the solid mass fraction increases, resulting in a reduced weight loss per unit time for the Si_3_N_4_/BN fiber-reinforced monolithic ceramics green body, thus decreasing the relative mass loss. The relative weight loss for the three solid mass fractions is 23.26 wt.%, 20.66 wt.%, and 17.18 wt.%, respectively.

#### 3.2.2. Defoaming Process for Green Bodies

**Figure 9:**
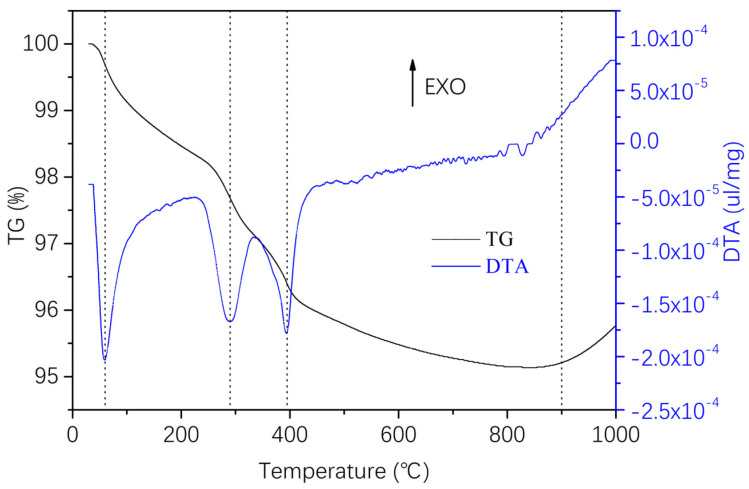
Thermogravimetric–differential thermal analysis (TG-DTA) of naturally air-dried Si_3_N_4_/BN fibrous monolithic ceramic green bodies.

Green bodies with organic content can expand and crack during direct sintering due to volatile substances. Even with minimal organic content in the spinning solution, uneven heating and decomposition product accumulation can cause cracks in larger components. Hence, oxidizing and eliminating the organic matter from the green body before sintering is essential. The defoaming rate is influenced by the volatilization rate of decomposition or oxidation products on the green body’s surface and the internal decomposition product’s diffusion rate. A sudden temperature spike can lead to a surge in volatile products, damaging the green body. Therefore, devising a defoaming process that considers the organic matter’s thermal decomposition characteristics is vital. Figure 9 presents the thermogravimetric–differential thermal analysis (TG-DTA) of naturally air-dried Si_3_N_4_/BN fibrous monolithic ceramics. The figure highlights the weight loss in Si_3_N_4_/BN fibrous monolithic ceramics from 100 °C to 900 °C. The weight loss between 100 °C and 250 °C is mainly due to bound water removal. A sharp slope in the TG curve between 250 °C and 400 °C indicates rapid sodium alginate decomposition. The TG curve’s slope mellows between 400 °C and 900 °C, possibly due to O_2_’s increased diffusion distance within the sample, slowing oxidation reactions. As the temperature continues to rise, the sample quality stabilizes, signifying the complete oxidation and removal of organic matter. Based on the TG analysis, the defoaming process for the green body is defined: the temperature is gradually increased to 300 °C at 1 °C/min and held for 1 h. It is then raised to 400 °C at the same rate and held for another hour. This method avoids decomposition product accumulation during initial defoaming, preserving the green body’s integrity. Finally, the temperature is increased to 900 °C at the same rate and held for 2 h, ensuring the binder’s complete oxidation and removal.

#### 3.2.3. Shrinkage Rate for Green Bodies

**Figure 10:**
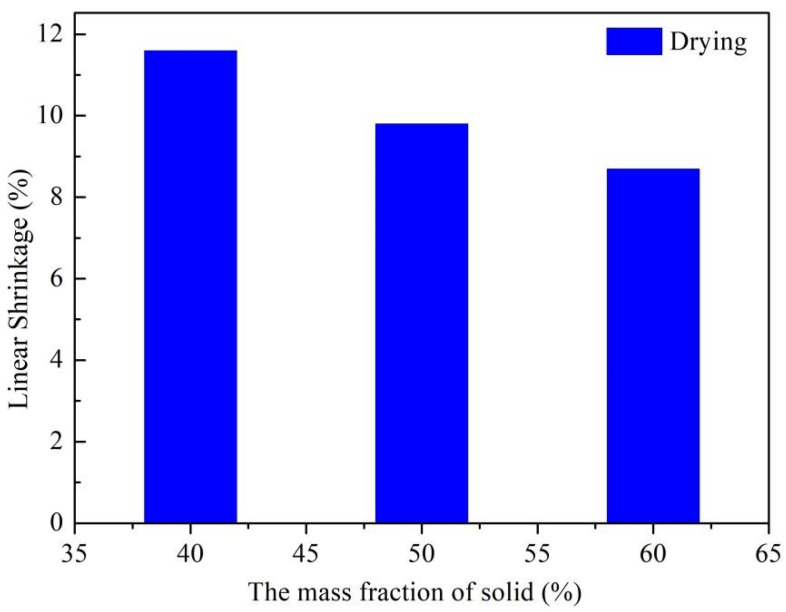
The effect of different mass fractions of solids on the linear shrinkage rate of billets during drying.

The final product’s dimensional accuracy hinges on the green bodies’ shrinkage rate post-drying and defoaming. Measuring the samples’ dimensional changes during each process provides insights for manufacturing large-sized components. However, the green body’s shrinkage rate after defoaming was not explored due to the project’s low organic binder content in the spinning solution. Figure 10 depicts the influence of varying solid mass fractions on the green body’s linear shrinkage rate during drying. The linear shrinkage rate of the green body post-drying drops from 11.6% to 8.7% as Si_3_N_4_ content increases. This decline is linked to the sample’s water proportion reduction as the solid mass fractions rise, leading to a denser solid-phase network. Consequently, less water removal results in a reduced shrinkage rate.

### 3.3. Mechanical Properties

**Figure 11:**
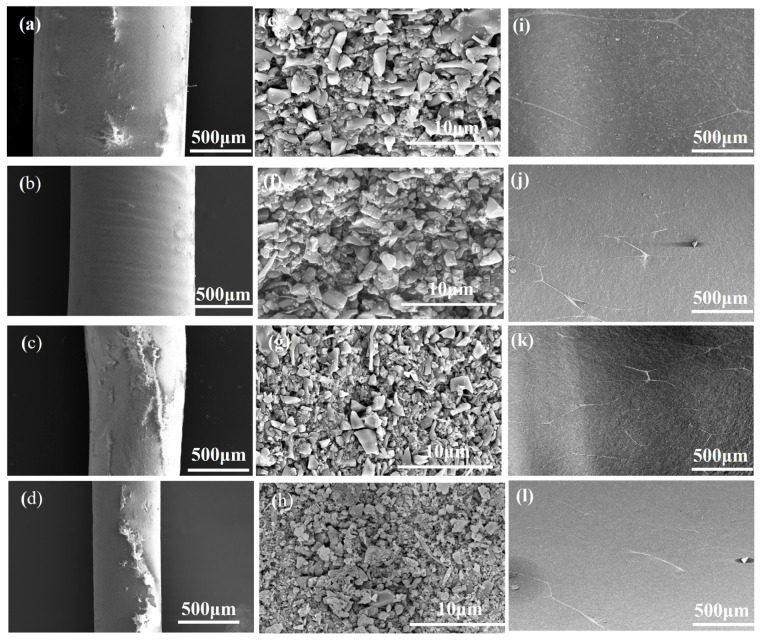
Microstructure of Si_3_N_4_ fibers before (**a**–**d**) and after (**e**–**h**) coating with BN, together with fibrous monolithic ceramics (**a**,**e**,**i**) Si_3_N_4_/BN-1, (**b**,**f**,**j**) Si_3_N_4_/BN-2, (**c**,**g**,**k**) Si_3_N_4_/BN-3, (**d**,**h**,**l**) Si_3_N_4_/BN-4.

Using injection molding technology, continuous Si_3_N_4_ fibers were extruded through injection needles with sizes of 14#, 16#, 18#, and 20#. This resulted in fibers of four different diameters, as illustrated in the microstructure shown in Figure 11a–d. After slurry treatment, the Si_3_N_4_ fibers were coated with a BN interface phase, and the resulting surface morphology after drying is displayed in Figure 11e–h. The BN coating resulted in an increase in fiber diameters to 0.42 mm, 0.7 mm, 0.9 mm, and 1.05 mm, accompanied by a rough fiber surface. Following assembly, degreasing, and sintering processes, Si_3_N_4_/BN fibrous monolithic ceramics were produced, designated as Si_3_N_4_/BN-1, Si_3_N_4_/BN-2, Si_3_N_4_/BN-3, and Si_3_N_4_/BN-4, corresponding to Si_3_N_4_/BN fibers with diameters of 1.05 mm, 0.9 mm, 0.7 mm, and 0.42 mm, respectively. Figure 11i–l shows the surface morphology of Si_3_N_4_/BN fibrous monolithic ceramics.

**Figure 12:**
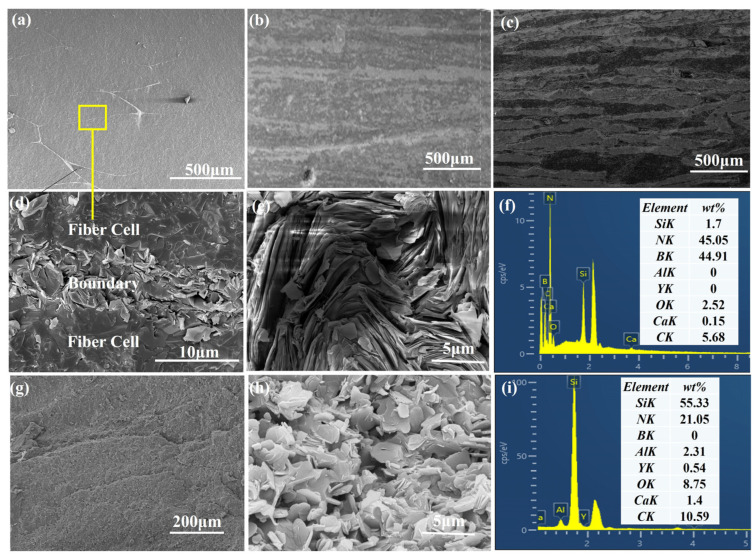
(**a**–**c**) Microstructures and morphologies of different surfaces; (**g**) fracture surfaces obtained from mechanical tests of Si_3_N_4_/BN fibrous monolithic ceramics; the enlarged morphology near cell boundary marked with yellow rectangle in (**a**); (**d**) low magnification; (**e**,**h**) high magnification; (**f**,**i**) major element distribution images.

Figure 12 provides a comprehensive view of the Si_3_N_4_/BN fibrous monolithic ceramics, showcasing their microstructure, morphology, polished surfaces, post-mechanical testing fracture surfaces, and elemental analysis. This figure highlights the composite’s anisotropic characteristics across three different polished surfaces, as shown in Figure 12a–c. Specifically, Figure 12a depicts the fiber cross-section as either an ellipse or a flattened hexagon, with surrounding boundaries forming an irregular mesh pattern. Due to varying pressing orientations during fabrication, the surfaces in Figure 12b,c display a layered look. The orientation in Figure 12b is perpendicular to the pressing direction, while in Figure 12c, it runs parallel. Further details on morphology and elemental distribution at cell boundaries can be found in Figure 12d–f,h,i, highlighting the differences between the fiber cells (*α*-Si_3_N_4_) and the cell boundaries (*h*-BN). Mechanical tests conducted at room temperature reveal that *h*-BN adopts a stratified flake structure, as demonstrated in Figure 12g.

**Figure 13:**
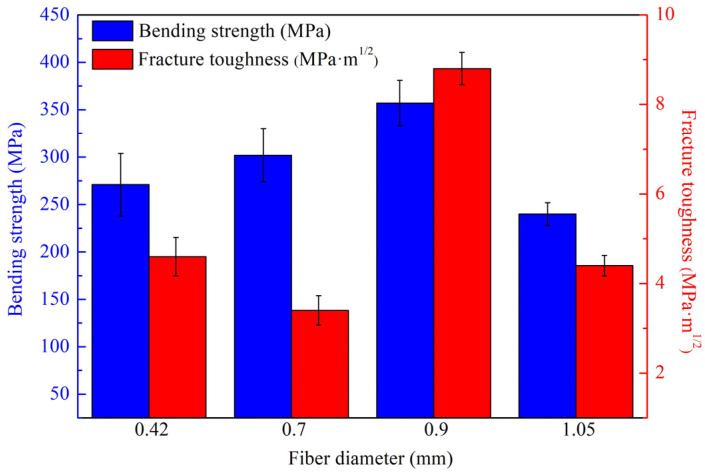
Mechanical properties of Si_3_N_4_/BN fibrous monolithic ceramics prepared from fibers of different diameters.

Figure 13 showcases the mechanical properties of Si_3_N_4_/BN fibrous monolithic ceramics reinforced with ceramic fibers of varying diameters. Si_3_N_4_/BN-4 ceramics, fabricated with ceramic fibers of 1.05 mm diameter, exhibited a bending strength of only 240 ± 12 MPa. This lower strength is due to the larger pores formed between the coarser fibers, which are challenging to fill during the hot-pressing processes [20]. Consequently, the material exhibits an increase in porosity, resulting in the deterioration of its mechanical properties. In contrast, Si_3_N_4_/BN-3 ceramics, using 0.9 mm diameter fibers, demonstrated an improved bending strength of 357 ± 24 MPa. However, when the fiber diameter was further reduced to 0.7 mm and 0.42 mm for Si_3_N_4_/BN-2 ceramics, a slight strength reduction was observed. This is because the smaller fiber diameter increases the density of weak interfaces in the composite, weakening the overall strength. The fracture toughness of Si_3_N_4_/BN-4, Si_3_N_4_/BN-3, Si_3_N_4_/BN-2, and Si_3_N_4_/BN-1 first increased and then decreased. Si_3_N_4_/BN-4 ceramics had a fracture toughness of only 4.4 ± 0.23 MPa·m^1/2^, whereas Si_3_N_4_/BN-3 ceramics exhibited a significant increase to 8.8 ± 0.36 MPa·m^1/2^, attributed to the higher number of weak interfaces due to the smaller fiber diameter. A subsequent reduction in the fiber diameter resulted in the fracture toughness of Si_3_N_4_/BN-2 and Si_3_N_4_/BN-1 ceramics decreasing to 3.4 ± 0.33 MPa·m^1/2^ and 4.6 ± 0.43 MPa·m^1/2^, respectively.

Table 3 compares the mechanical properties of Si_3_N_4_/BN materials prepared with various fiber diameters in this study to the findings reported in the literature. The mechanical properties of Si_3_N_4_/BN materials are consistently subpar regardless of the sintering method employed. However, introducing a fibrous monolithic structure to Si_3_N_4_/BN materials has partially enhanced their mechanical properties. On the downside, the debinding process, executed in an inert atmosphere, negatively affects wave transmission performance. Additionally, the incorporation of *β*-Si_3_N_4_ and SiC whiskers to enhance the mechanical properties of Si_3_N_4_/BN fibrous monolithic ceramics significantly affects their wave transmission performance. In our research, the debinding process for Si_3_N_4_/BN fibrous monolithic ceramics takes place in ambient air. The carbon element content in Si_3_N_4_/BN fibrous monolithic ceramics was measured to be 0.82% using an elemental analyzer. This composition does not notably degrade their mechanical properties. The best mechanical performance was achieved with a fiber diameter of 0.90 mm, markedly outperforming other sintering techniques.

## 4. Conclusions

This study introduces a wet-spinning–hot-pressing strategy for crafting Si_3_N_4_/BN fibrous monolithic ceramics. By incorporating a high concentration of sodium alginate, crosslinking is enhanced, leading to a boost in the slurry’s modulus and viscosity. On the other hand, a rise in solid content intensifies van der Waals interactions, which could cause potential needle blockage. To understand this better, we adjusted the mass fractions of sodium alginate and solid content, examining the rheological properties of the spinning slurry. We also explored how varying the Si_3_N_4_/BN fiber diameter impacts the ceramics’ microstructure and mechanical properties. Notably, the storage modulus of the P2 sample was approximately 205 Pa, with a yield stress (*τ*_0_) of 455 Pa. These values are ideal for extrusion. exhibiting favorable self-supporting capability, making it apt for wet spinning. The resulting Si_3_N_4_/BN fibrous monolithic ceramics exhibited a carbon content of 0.82%, a three-point bending strength of 357 ± 24 MPa, and a fracture toughness of 8.8 ± 0.36 MPa·m^1/2^. This investigation offers valuable insights on producing high-performance Si_3_N_4_/BN composite ceramics utilizing hot-pressing technology.

## Figures and Tables

**Table 1:** Proportions of initial powders.

*α*-Si_3_N_4_ (wt.%)	Y_2_O_3_ (wt.%)	Al_2_O_3_ (wt.%)
95	3.57	1.43

**Table 2:** Compositions of spinning solution.

Samples	Initial Powders (wt.%)	Deionized Water (wt.%)	Sodium Alginates (wt.%)
P1	40	58.2	1.8
P2	50	48.75	1.25
P3	60	38.8	1.2
P4	50	49	1
P5	50	48.5	1.5

**Table 3:** Comparison of the bending strength and fracture toughness between samples sintered in this work with those reported in the literature.

Composition (wt.%)	Sintering Method	Bending Strength (MPa)	Fracture Toughness (MPa·m^1/2^)	References
Si_3_N_4_/BN	gel cast	128	2.0	[21]
Si_3_N_4_/BN	gas pressure sintering	215	4.31	[22]
Si_3_N_4_/BN	pressureless sintering	243	2.75	[23]
Si_3_N_4_/BN	gel cast/pressureless sintering	190.1	4.16	[24]
Si_3_N_4f_/BN/Si_3_N_4_	CVI	98 ± 9	3.7 ± 0.3	[25]
3D Si_3_N_4f_/BN/Si_3_N_4_	PIP	191 ± 13	5.8 ± 0.5	[26]
Si_3_N_4_/BN	reaction bonding technology	160	—	[27]
Si_3_N_4_/BN	wet spinning/hot pressing/inert atmosphere debinding	380	10	[11,12]
Si_3_N_4_/BN/SiC*_f_*	wet spinning/hot pressing/inert atmosphere debinding	705.4	23.95	[13,14,15]
Si_3_N_4_/BN/*β*-Si_3_N_4_	wet spinning/hot pressing/inert atmosphere debinding	530.6	17.16	[16,17]
Si_3_N_4_/BN-1 (0.42 mm)	wet spinning/hot pressing/air atmosphere debinding	271 ± 33	4.6 ± 0.43	This work
Si_3_N_4_/BN-2 (0.70 mm)	wet spinning/hot pressing/air atmosphere debinding	302 ± 28	3.4 ± 0.33	This work
Si_3_N_4_/BN-3 (0.90 mm)	wet spinning/hot pressing/air atmosphere debinding	357 ± 24	8.8 ± 0.36	This work
Si_3_N_4_/BN-4 (1.05 mm)	wet spinning/hot pressing/air atmosphere debinding	240 ± 12	4.4 ± 0.23	This work

## Data Availability

The data used to support the findings of this study are available from the corresponding author upon request.
